# Identification of G protein-coupled receptors required for vitellogenin uptake into the oocytes of the red flour beetle, *Tribolium castaneum*

**DOI:** 10.1038/srep27648

**Published:** 2016-06-09

**Authors:** Hua Bai, Subba Reddy Palli

**Affiliations:** 1 Department of Genetics, Development, and Cell Biology, Iowa State University, Ames, IA 50011, USA; 2 Department of Entomology, University of Kentucky, Lexington, KY 40546-0091, USA

## Abstract

Previous studies suggested that a membrane receptor might be involved in mediating vitellogenin (Vg) uptake and juvenile hormone (JH)-regulated remodeling of follicular epithelium (also called ‘patency’). G protein-coupled receptor (GPCR) family is one of the largest membrane receptor protein families and controls many key physiological processes. To investigate the role of GPCRs in insect reproduction and juvenile hormone-regulated Vg uptake, we performed a comprehensive RNA interference (RNAi) screen targeting GPCRs in the red flour beetle, *Tribolium castaneum*. Out of 112 GPCRs tested, knockdown of 41 GPCRs resulted in a reduction in fecundity. Interestingly, RNAi against two GPCRs (a Rhodopsin-like receptor and a Dopamine D2-like receptor) led to a significant reduction in Vg accumulation in developing oocytes. Functional assays of these two GPCRs showed that JH triggers a dose-dependent inhibition of intracellular cAMP levels in HEK293 cells expressing *Tribolium* Dopamine D2-like receptor. These data suggest that Dopamine D2-like receptor plays crucial roles in regulating Vg uptake and is a promising candidate membrane receptor mediating JH regulation of patency in the red flour beetle.

Juvenile hormone (JH) is one of the major hormones that regulate insect development, reproduction and behavior. In adult insects, JH acts on many tissues including fat body, gonads, accessory glands, flight muscle and nervous system^1^. In female insects, JH mainly regulates vitellogenin (Vg) gene expression in fat body^2^,^3^,^4^,^5^ and uptake of Vg or yolk protein (YP) into the oocyte^6^. Pratt and Davey (1972)^6^ have shown that JH can induce ‘patency’ (increase in intercellular spaces between follicle cells to allow uptake of Vg into oocytes) in isolated ovaries of *Rhodnius prolixus*. Patency seems to be a universal phenomenon during insect vitellogenesis, as it has been reported from many insect species including *Blattella germanica*^7^, *Locusta migratoria*^8^, *Tenebrio molitor*^9^, *Hyalophora cecropia*^10^, *and Heliothis virescens*^11^. Furthermore, allatectomized female *Periplaneta americana* failed to uptake Vg, which was rescued by implanting corpora allata (CA) or injecting a JH analog^12^. In *apterous* mutant fruit fly where JH biosynthesis is reduced, Vg uptake by ovaries is blocked, regardless the normal synthesis of Vg^13^. Methoprene, a JH analog, can restore yolk deposition in the *apterous* mutant. Interestingly, Vg protein was detected in diapausing fruit flies with low levels of JH. However, no Vg protein was taken up into oocytes^14^. These studies suggest that JH signaling plays a key role in Vg uptake into oocytes. However, the underlying mechanisms through which JH regulates this process remain unclear.

Previous studies in *R. prolixus* suggest that protein kinase C and Na^+^/K^+^ ATPase cascades might be involved in the regulation of patency and Vg uptake. A 100 kDa protein, putative α-subunit of Na^+^/K^+^ ATPase, was isolated from the follicle membranes of *R. Prolixus*^15^,^16^. A rapid decrease in intracellular Ca^2+^ was observed in locust follicle cells after exposure to JH III^1^. The Ca^2+^ binding protein, calmodulin, was expressed in a JH-dependent manner during ovarian development in *B. germanica*^17^. In *H. virescens*, JH II and JH III-regulated patency is associated with voltage-dependent Ca^2+^ channels, while JH I regulated patency appears to be Ca^2+^ independent^18^. Pszczolkowski *et al*.^11^ found that JH I and JH III evoked patency in *H. virescens* through different second messenger pathways, cAMP-dependent and inositol triphosphate/diacylglycerol signal pathway, respectively. These studies suggest that JH I may act on follicular epithelial cells via activation of G_s_ protein-coupled receptors in *H. virescens*. By using a photoaffinity analog of JH III, [^3^H]EFDA, a 35 kDa protein was isolated from the membranes of locust follicle cells^19^. However, the function of this protein in JH-regulated patency and Vg uptake remains unknown. Recent studies in mosquito reported that activation of the PLC pathway by JH resulting in an increase in the intracellular concentrations of DAG, IP3, and calcium and enhanced binding of Met–Taiman complex to JH response elements (JHREs)^20^. These studies suggest that JH-regulated patency and Vg uptake are regulated through a membrane receptor, possibly a G protein-coupled receptor (GPCR). However, the nature of this receptor is still an enigma.

Since accumulated evidence suggests that GPCRs are the potential candidates involved in JH-induced nongenomic action during insect vitellogenesis, we carried out a comprehensive RNA interference (RNAi) screen to identify the candidate GPCR(s) that is required for Vg uptake in the red flour beetle, *Tribolium castaneum*. GPCRs are seven-transmembrane receptor proteins that sense external signals and activate a variety of intercellular pathways. GPCR family is considered as the largest integrate membrane receptor family, and it can be found in almost all eukaryotes^21^. About 16,000 GPCR sequences from various organisms have been deposited in GPCRDB (www.gpcr.org/7tm)^22^. To date, about 200 GPCRs were identified in *Drosophila melanogaster*^23^ and 276 GPCRs in *Anopheles gambiae*^24^. Also, 35 neuropeptide receptor genes and 19 biogenic amine receptor genes were identified in the honey bee *Apis mellifera*^25^. In the recently sequenced *Tribolium* genome, we and others have identified two Rhodopsin receptors and 111 non-sensory GPCRs including 74 Rhodopsin-like GPCRs, 19 Secretin receptor-like GPCRs, 11 Metabotropic glutamate receptor-like GPCRs, and 7 Atypical GPCRs^26^,^27^. Through our RNAi screen, we identified 41 GPCRs that play important roles in female fecundity. Surprisingly, only two of them, a Rhodopsin-like receptor and a Dopamine D2-like receptor, are crucial for Vg uptake. We then functionally characterized these receptors in HEK293 cells and showed that the cells expressing Dopamine D2-like receptor were able to respond to JH stimulation in a dose-dependent manner. Taken together, our results suggest that Dopamine D2-like receptor is one of the key receptors that mediate JH regulation of patency in *T. castaneum*.

## Materials and Methods

### Beetle rearing and staging

*T. castaneum* Strain GA-1^28^ were reared on organic wheat flour containing 10% yeast at 32 °C and 40% humidity in complete darkness condition. Female and male pupae were sexed based on genital papillae and placed in separate cups before adult eclosion.

### Double-stranded RNA (dsRNA) synthesis

Primers containing GPCR gene sequences and T7 polymerase promoter (TAATACGACTCACTATAGGG) at the 5′-end of both the forward primer and reverse primer were used to amplify a 200–600 bp region of the gene coding for each GPCR as reported previously^27^. The PCR product was used as a template for dsRNA synthesis using the Ambion MEGAscript transcription kit (Ambion, Austin, TX). DsRNA was purified using phenol/chloroform extraction followed by ethanol precipitation and dissolved in nuclease-free water to a concentration of 3–5 μg/μl. The quality of dsRNA was checked by running on an agarose gel and the concentration was measured using NanoDrop1000 spectrophotometer (Thermo Fisher Scientific Inc., Waltham, MA).

### DsRNA Microinjection

Newly emerged female beetles were anesthetized using ether vapors for 5 min, and then placed in double-sided sticky tape over a glass slide. About 50–100 nl of dsRNA was injected into each female on the lateral side of the second or third abdominal segment using an aspirator tube assembly (Sigma, St. Louis, MO) fitted with a 3.5 inch glass capillary tube (Drummond, Broomall, PA) pulled by a needle puller (Model P-2000, Sutter Instruments Co., Novato, CA). Injected females were allowed to recover for one hour at room temperature and then transferred to 30 °C incubator. Control beetles were injected with dsRNA for the *malE* gene (maltose-binding protein) from *Escherichia coli*.

### Screen for fecundity and larva hatch rate

DsRNAs for 112 GPCRs were injected into the adult females within 12 h after emergence and the dsRNA injected females were then mated with uninjected virgin males at three-day post injection (2~3 pairs were grouped in one plastic cup containing wheat flour and yeast). The eggs were separated from flour and transferred to a new cup at one week and two weeks after mating, respectively. Female fecundity was presented as the number of eggs laid from each pair per week. The larva hatch rate was calculated as the percentage of the number of hatched larvae versus the total number of the eggs laid. The number of eggs laid by each malE control female per week is about 29.71 ± 3.58 (Table S1), while the larva hatch rate is about 74.93 ± 4.03% (Table S2).

### Enzyme-linked immunosorbent assay (ELISA)

An ELISA method was developed for quantifying Vg content from extracted hemolymph and ovary samples. Hemolymph from five females was extracted by decapitation and centrifugation to collect supernatant containing serum proteins. Ovaries dissected from five females were homogenized and the supernatant was collected after centrifuge. 100 μl of diluted hemolymph or ovary samples were coated directly onto 96 well easy wash EIA/RIA plate (Corning Incorporated, Corning, NY) and incubated overnight at room temperature. Serial dilutions of recombinant *T. castaneum* Vg protein (TcVg, produced in our previous study^29^) were used to generate a standard curve. Following incubation, coated protein samples were discarded and the plate was washed three times with PBS-Tween (0.05% polyoxyethylene-20 sorbitan monolaurate) (Sigma, St. Louis, MO). All wells then were blocked in EIA buffer (10 mM Na2HPO4, 3 mM NaH2PO4, 150 mM NaCl, 1 mM NaEDTA·2dH2O, 0.2% NaN_3_, 1 mg/ml BSA) for two hours at room temperature. After blocking and three times washing with PBS-Tween, 100 μl of anti-TcVg antibody produced in our recent study^29^ (1: 1500 in EIA buffer) were added to each well except for the blank well. Following one hour incubation, all wells were washed three times with PBS-Tween and 100 μl of anti-rabbit IgG horse radish peroxidase-linked antibody from donkey (GE Healthcare UK Limited, England) (1: 2500 in EIA buffer) were added to all wells and incubated at room temperature for 1 h. Then the plates were washed and color reaction was performed using 100 μl TMB solution (3,3′,5,5′-teramethylbenzidine) (American Qualex Antibodies, San Clemente, CA). The absorbance was recorded at 595 nm using a Thermo Labsystems Multiskan Plus spectrophotometer (Thermo Electron Corporation, Waltham, MA).

### Western blot

An equal amount (10 μg) of protein from each sample was denatured at 95 °C for 5 min in 1× loading buffer. Denatured protein samples were loaded and separated on 10% SDS-polyacrylamide gels and transferred to nitrocellulose membranes (Bio-Rad, Hercules, CA) using a Trans-blot semidry transfer cell (Bio-Rad). Membranes were blocked in 5% blotto solution (BLOT-Quickblocker; GENO Technology, Inc. St. Louis, MO) in TTBS buffer for 1 hr, and incubated with 1:5000 anti-TcVg1 antibody produced in rabbit overnight at 4 °C. Membranes were washed in TTBS and incubated with 1:5000 goat anti-rabbit IgG conjugate with alkaline phosphatase (Sigma, St. Louis, MO) for 1 hr. After washing with TTBS and alkaline phosphatase substrate buffer, recombinant proteins were visualized by exposing the membrane to the mixture of nitro blue tetrazolium and 5-bromo-4-chloro-3-indolyl phosphate (Sigma, St. Louis, MO).

### Follicle length measurement and staging

Ovaries at various stages were dissected in 1× PBS and the length of the most developed primary follicle from each ovary was measured using an ocular micrometer. We also classified each ovary into two distinct stages, previtellogenic stage and vitellogenic stage. The major difference between these two stages is the presence of vitellogenin (yolk protein) in the ovary, which is seen as dark content inside each developing oocyte.

### Patency assay

Ovaries were dissected in 1× PBS and 3–5 ovarioles were removed from each ovary. After washing 3 times in Ex-cell 420 medium (SAFC Inc., Haverhill, MA), the ovarioles were incubated in centrifuge tubes containing 500 μl medium for 1 hr at 30 °C in order to remove endogenous hormones. Then ovaries were transferred to borosilicate incubation tubes containing 50 μl medium with either DMSO solvent or hormones (JH III and 20E, 20-hydroxyecdysone, Sigma, St. Louis, MO). Ovaries were incubated at 30 °C for 2 hr. Ovarioles were then placed in a drop of 1% Evans Blue for 10 sec on a cavity slide. After washing with medium, ovaries were observed under an inverted microscope and photographed. The length of randomly selected follicular epithelial cells were measured using NIH ImageJ program. Patency index was calculated using the formula [d3/(d1 + d2)] X 100 described by Pszczolkowski *et al*.^11^, where d1 and d2 are lengths of neighboring cells and d3 is the width of the intercellular space between cells.

### Scanning electronic microscopy (SEM)

Ovaries were dissected in 1× PBS and immediately transferred to 1% glutaraldehyde in PBS for 1–2 hr at 4 °C. Then, ovaries were washed with distilled water for 5 min and dehydrated through ethanol solutions of 70%, 85%, 95% and 100%, with 5 min in each. Ovaries were transferred to HMDS (1,1,1,3,3,3 hexadimethyldisilazane) for 5 min and mounted on stainless steel stubs with double sticky tabs after air dry at room temperature^30^. The mounted ovaries were then coated with gold using Hummer VI Sputtering System at plasma discharge rate of 10 milliamperes for 180 s. Scans were performed with a Hitachi S-800 Scanning Electron Microscope (Hitachi, Japan) at 10 kV and 10 milliamperes. Images were documented using Evex Nanoanalysis and Digital Imaging software (Evex Analytical version 2.0.1192).

### cAMP reporter assay

The predicted open reading frame (ORF) for candidate GPCR genes were cloned into pCDNA3.1 expression vector (Invitrogen, Carlsbad, CA). Human embryonic kidney (HEK) 293 cells were cultured in DMEM medium supplied with 10% FBS at 37 °C with 5% CO_2_. Cells were plated onto a white opaque 96-well plate (2~5 × 10^4^ per well) 8 hours prior to transfection. Cells were then transiently transfected with pCDNA3.1/GPCR constructs together with a human Gα15 expression construct and a 6 × CRE-luciferase reporter construct (Kindly provided by Dr. Alan S. Kopin, Molecular Pharmacology Research Center, Tupper Research Institute, Tufts–New England Medical Center) using Lipofectamine 2000 (Invitrogen, Carlsbad, CA). Thirty-six hours post-transfection, cells were exposed to ligands (JH III or DA, dopamine hydrochloride, Sigma, St. Louis, MO) and 1 μM of forskolin for 4 hr in serum-free medium. Cells then were lysed using Bright-Glo™ Luciferase Assay reagent (Promega Corporation, Madison, WI) for 2 min in the dark. The luciferase activity was quantified using a SpectraMax M5 microplate reader (Molecular Devices, Sunnyvale, CA, USA). At least six replicates were used for each treatment.

### Statistical analysis

GraphPad Prism 6 (GraphPad Software, La Jolla, CA) was used for statistical analysis. To compare the mean value of GPCR RNAi versus that of control, one-way ANOVA analysis was performed (Dunnett’s test included for comparison of multiple means). The comparison between hemolymph Vg content and ovarian Vg among GPCR RNAi groups was analyzed by two-way ANOVA (Uncorrected Fisher’s LSD multiple comparison included).

## Results

### Large-scale RNAi screen to identify GPCRs involved in female fecundity

**Figure 1:**
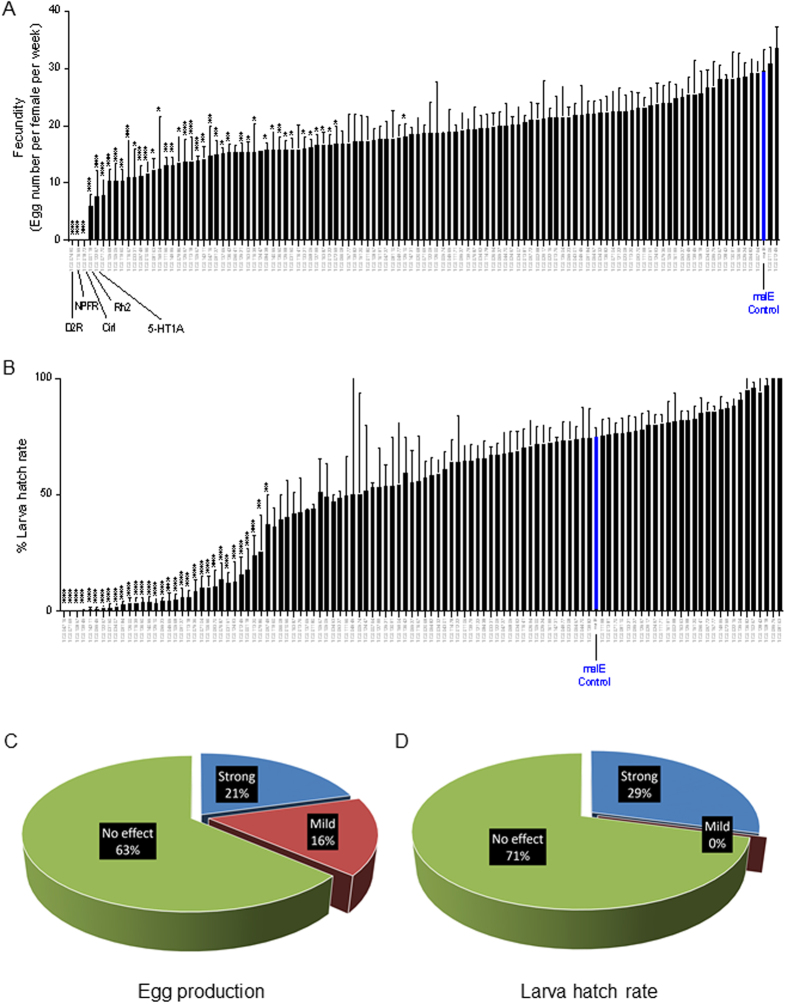
RNAi screen identified GPCRs involved in female fecundity and larva hatch rate in *T. castaneum*. (**A**) Fecundity of females injected with dsRNAs targeting 112 GPCRs and malE control. Fecundity is presented as egg number per female per week. (**B**) Larval hatch rate from eggs laid by RNAi females. Hatch rate is calculated as the percentage of the number of hatched larvae versus the total number of the eggs laid. Each bar represents mean ± SE of 4~10 cohorts, 12~20 pairs of beetles. Statistical significance between the mean value of GPCR RNAi versus that of malE control is assessed by one-way ANOVA analysis, followed by Dunnett’s multiple comparisons (***p < 0.001, **p < 0.01, *p < 0.05). malE data were marked as blue bars. (**C**) The pie chart to show the percentage of RNAi effects on fecundity. (**D**) The pie chart to show the percentage of RNAi effects on larva hatch rate. (Strong effect p < 0.01, Mild effect p < 0.05, No effect p > 0.05).

To identify candidate GPCRs involved in reproduction, a large-scale RNAi screen was performed by knocking down the expression of 112 GPCRs in female *T. castaneum*, including two Rhodopsin-like receptors and 110 non-sensory GPCRs identified in our recent study^27^. Since Vg uptake is a vital process for oocyte development, female fecundity and embryonic development, we carried out our first screen by examining the effects of GPCR RNAi on female fecundity and larva hatch rate. Out of 112 GPCRs tested, RNAi against 23 GPCRs (21%) resulted in strong reduction in egg production (p < 0.01), while knockdown of 18 GPCRs (16%) showed mild effects on egg production (p < 0.05) (Fig. 1A,C, Table S1) (Statistical significance was analyzed by one-way ANOVA, followed by Dunnett’s multiple comparisons). Notably, females injected with dsRNAs for three GPCRs did not lay any eggs. These GPCRs are TC007490/D2R (Dopamine 2-like receptor), TC011655/NPFR (Neuropeptide F receptor), and TC001872/Cirl (Latrophilin-like receptor) (Fig 1A, Table S1). Also, knockdown of the expression of three genes, TC000118/Rh2 (One of the two Rhodopsin-like receptors), TC012297/5-HT1A (Serotonin receptor 1A-like), and TC007170/CapaR (capa receptor-like) result in ~75% reduction in the egg production (Fig. 1A, Table S1).

In *T. castaneum*, dsRNA delivered into female beetles can result in knockdown of zygotic genes in offspring embryos (called ‘parental RNAi’)^31^. This allowed us to further investigate the potential roles of GPCRs in the regulation of embryonic development. About 29% of GPCR dsRNAs injected to female beetles led to a strong reduction in larva hatch rate (p < 0.01) (Fig. 1B,D, Table S2). Interestingly, some GPCRs are specifically involved in embryonic development but play less or no roles in female fecundity. For example, TC013945/CcapR (Crustacean cardioactive peptide receptor), TC007536 (Sulfakinin receptor, TC007104 (Diuretic hormone receptor), TC010656 (Methuselah-like receptor), and TC009370 (Methuselah-like receptor).

### RNAi screen to identify GPCRs involved in Vg uptake

**Figure 2:**
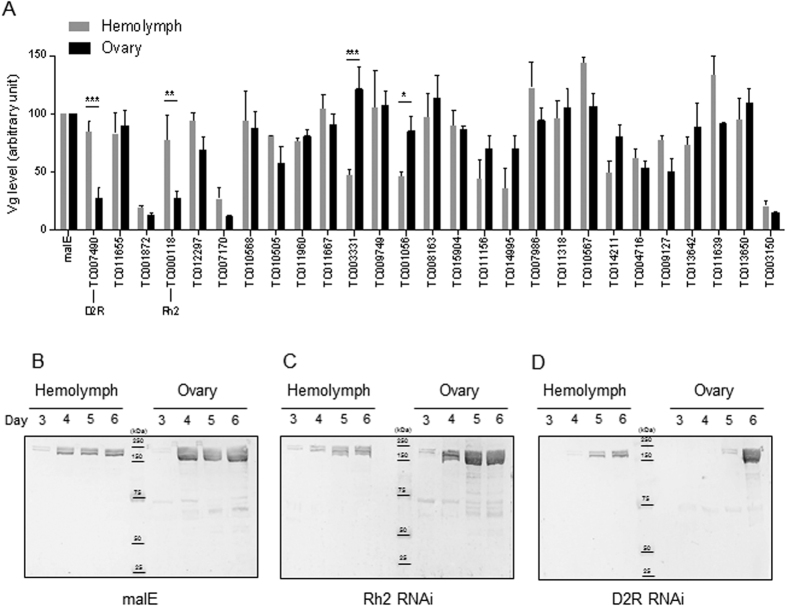
RNAi screen identified GPCRs involved Vg uptake in *T. castaneum*. (**A**) Vg content measured by ELISA from hemolymph and ovary samples in 27 GPCR RNAi beetles. Each bar represents mean ± SE of 3~9 independent trials. Statistical significance is assessed by two-way ANOVA analysis followed by uncorrected Fisher’s LSD multiple comparison (***p < 0.001, **p < 0.01, *p < 0.05). (**B**–**D**) Western blots showed Vg protein levels in hemolymph and ovary samples isolated from females injected with dsRNAs for malE, TcRh2 and TcD2R. Four ages of females were examined, day 3~6 PAE. Two distinct bands (~200 kDa and ~170 kDa) are observed. Vg levels increase with age, which are blocked (or delayed) by knocking down TcRh2 and TcD2R.

**Figure 3:**
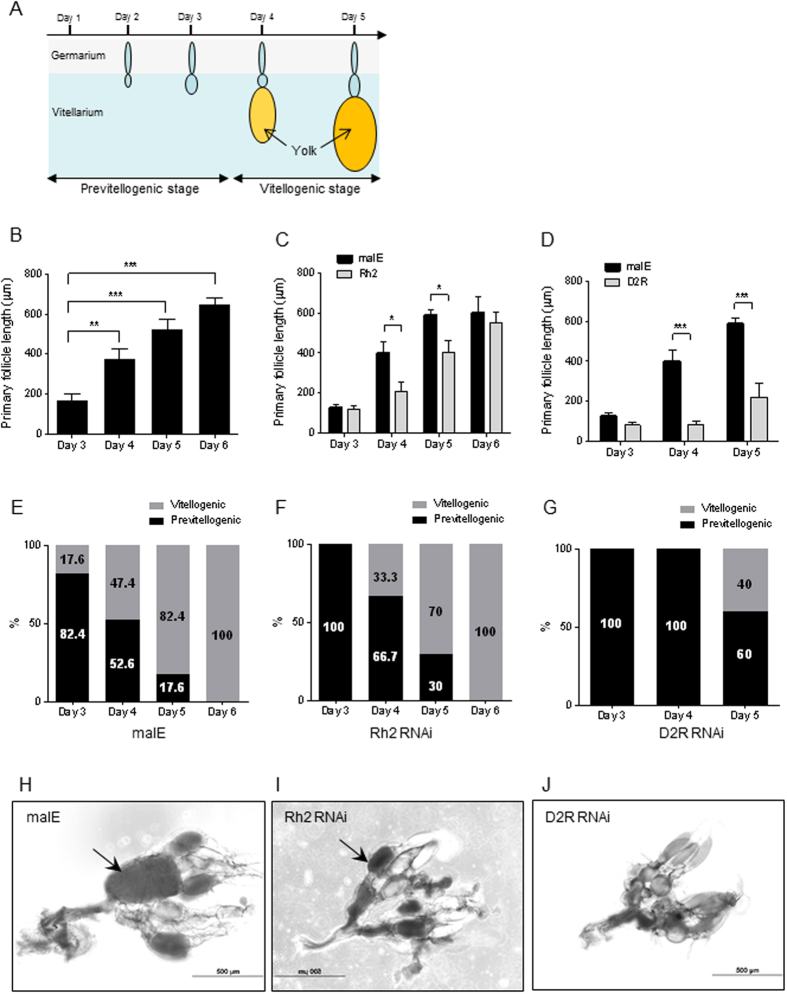
Inhibition of ovarian development in TcD2R RNAi beetles. (**A**) Schematic representation to show ovarian growth and two distinct ovarian stages at various ages. (**B**) Measurement of primary follicle length of ovaries dissected from malE females. (**C**) Comparison of the primary follicle length between malE and TcRh2 RNAi females. (**D**) Comparison of the primary follicle length between malE and TcD2R RNAi females. Each bar represents mean ± SE of 7~19 isolated ovaries. Statistical significance is assessed by one-way ANOVA analysis, followed by Dunnett’s multiple comparisons (***p < 0.001, **p < 0.01, *p < 0.05). (**E**–**G**) The percentage of two ovarian stages (previtellogenic and vitellogenic) Characterization of the ovarian stage in females injected with dsRNAs for malE, TcRh2 and TcD2R. (**H**–**J**) Light microscope images to show the ovaries isolated from females injected with dsRNAs for malE, TcRh2 and TcD2R on day 5 PAE. The dark arrow indicates oocytes that contain yolk and lipid droplets.

To directly examine the role of candidate GPCRs in the regulation of Vg uptake, a polyclonal antibody was generated against one of the two Vg genes of *T. castaneum* (as reported in our recent study)^29^. The specificity of the antibody is relatively high and the antibody only recognized two bands (~200 kDa and ~170 kDa) corresponding to two subunits of Vg protein (Fig. 2B–D and data from the previous study^29^) in both hemolymph and ovary samples. Using this antibody, we observed a sharp increase of Vg content presented in the hemolymph and the isolated ovaries on day 4 post adult eclosion (PAE), which is correlated to the timing of ovarian development and vitellogenesis *in vivo* (Fig. 3B,E). Accordingly, in the Vg uptake screen, hemolymph and ovary samples were collected on day 5 PAE when the majority of ovaries (82.4%) showed yolk deposition in the ovaries in control insects.

We selected 27 GPCR candidates whose RNAi resulted in a strong reduction (p < 0.01) in female fecundity based our first screen (Fig. 1A, Table S1) to screen for GPCRs that are involved in Vg uptake. Out of 27 GPCRs tested, RNAi against half of the GPCRs led to a decrease in either circulating or ovarian Vg levels (Fig. 2A). In some cases, GPCR knockdown resulted in a reduction of Vg levels in both hemolymph and ovaries, such as RNAi against TC001872/Cirl, TC007170/capaR and TC003150/sNPFR. Knockdown of two GPCRs (TC003331 and TC001056) led to a decrease in circulating Vg, but not ovarian Vg content, suggesting these GPCRs are involved in Vg synthesis, instead of Vg uptake.

Only two GPCRs (TC007490/D2R and TC000118/Rh2) were identified as promising candidates for Vg uptake, as RNAi against these GPCRs results in a significant decrease in ovarian Vg content when compared to the effects on circulating Vg level (Fig. 2A). As mentioned earlier, Vg uptake (as well as Vg synthesis) increases dramatically with the age of the beetles. We, therefore, examined the age-dependent Vg level in TcRh2 and TcD2R RNAi insects. Interestingly, we observed a delay of Vg accumulation in ovaries of both GPCR knockdown beetles, especially in TcD2R RNAi beetles (Fig. 2B–D). It should be noted that RNAi against TcRh2 and TcD2R also led to a delay in Vg release into the hemolymph. However, it is clear that TcD2R RNAi blocks Vg uptake on day 5 PAE when circulating Vg in TcD2R RNAi insects has almost reached the control levels (Fig. 2D).

### Inhibition of ovarian development in TcD2R RNAi beetles

To further investigate *in vivo* functions of two identified GPCRs and their potential roles in ovarian development, we examined the morphology of ovaries isolated from RNAi beetles at various times after dsRNA injection. The ovaries of *T. castaneum* contain 7–8 telotrophic finger-like ovarioles. Each ovariole is composed of two distinct structures: the germarium that contains nurse cells and developing follicle cells; the vitellarium that contains the primary oocytes surrounded by mature follicle cells (Fig. 3A). Unlike polytrophic type ovaries found in fruit flies and mosquitoes, ovarian development is asynchronous in *T. castaneum*. Although each ovary contains ovarioles at different developmental stages, the majority of the basal oocytes in ovarioles develop in a similar fashion. Therefore, the most developed oocyte in each ovariole was used for staging and measurements.

As shown in Fig. 3B, the length of primary oocytes steadily increases during the ovarian maturation and reach the maximum size of 600–700 μm by day 6 PAE. In the meantime, yolk and lipid droplets begin to appear on day 3 PAE and accumulation of yolk or Vg in the primary oocytes reach the maximum levels by day 6 PAE (data not shown). However, if only a few primary oocytes reach maximum yolk deposition, it is hard to use western blot to differentiate them from those with many oocytes that have mid-level of yolk deposition, due to the asynchronous development of each ovariole within the same ovary. Interestingly, a slow growth of primary follicle was observed in both TcRh2 and TcD2R RNAi females (Fig. 3C,D). Notably, on day 5 PAE the follicle length in malE control females was increased by 4.7-fold in two days, while there was only a 2.7-fold increase in TcD2R knockdown females (Fig. 3D).

For staging purpose, we defined the primary oocyte without yolk as the previtellogenic oocyte and the oocyte with yolk as the vitellogenic oocyte. About 17.6% of primary oocytes in the ovaries dissected from day 3 PAE control insects contained yolk protein. With time more and more ovaries contained vitellogenic oocytes and by day 6 PAE all of the ovaries isolated from control females contained vitellogenic oocytes (Fig. 3E,H). The trend of ovarian development in TcRh2 RNAi females is very similar to the control (Fig. 3F,I). However, there were more ovaries dissected from TcD2R RNAi females with undeveloped, previtellogenic oocytes (Fig. 3G,J). These results suggest that a block in Vg uptake by TcD2R RNAi may have caused a delay in ovarian growth and development.

### *In vitro* functional assay links TcD2R to JH signaling

**Figure 4:**
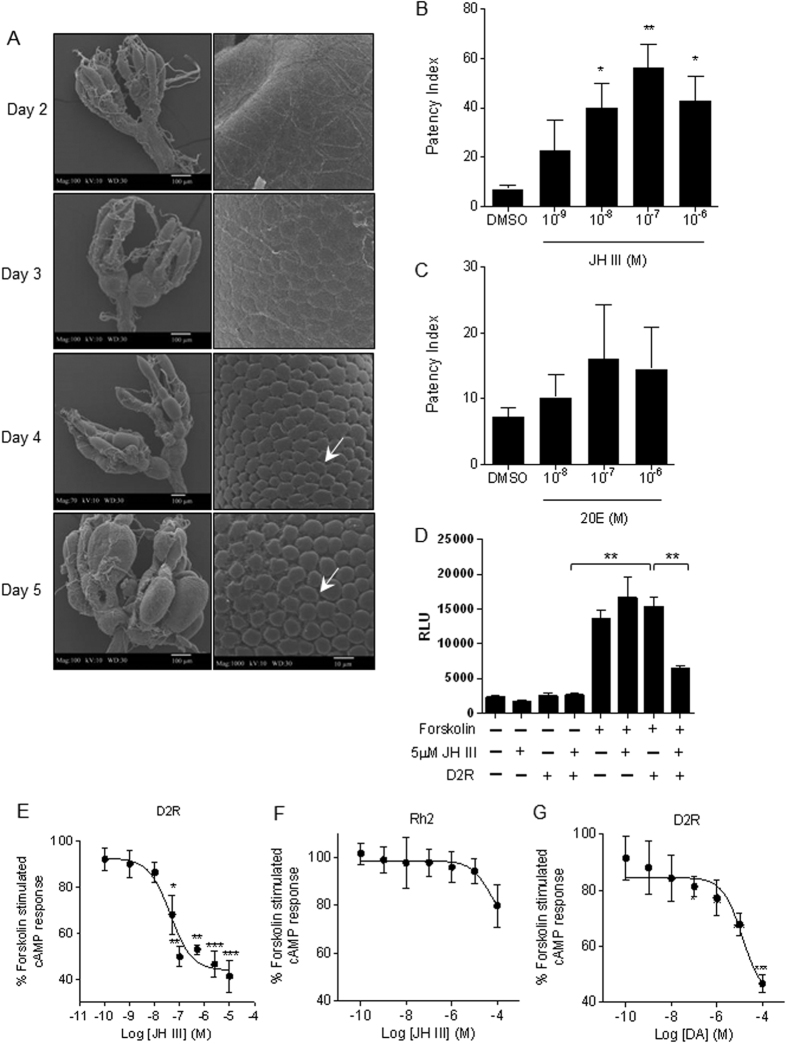
*In vitro* functional assay links TcD2R to JH is signaling. (**A**) Scanning electronic microscopy showing ovarian development and patency in female *T. castaneum.* Images on the right show the follicular epithelium without or with patency (arrow) at high magnification. (**B**) JH induces patency in isolated ovaries in a dose-dependent manner. (**C**) 20E shows no apparent induction on patency. Each bar represents mean ± SE of 3–5 ovarioles isolated from four female on day 3 PAE. Statistical significance is assessed by one-way ANOVA analysis, followed by Dunnett’s multiple comparisons (***p<0.001, **p < 0.01, *p < 0.05). (**D**) cAMP reporter assay shows that JH III inhibits forskolin-triggered cAMP levels in HEK293 cells expressing TcD2R. (**E**) Dose-response curves for the effect of JH III on forskolin-induced cAMP levels in HEK293 cells expressing TcD2R. (**F**) Dose-response curves for the effect of JH III on forskolin-induced cAMP levels in HEK293 cells expressing TcRh2. (**G**) Dose-response curves for the effect of dopamine (DA) on forskolin-induced cAMP levels in HEK293 cells expressing TcD2R. Base cAMP level induced by 4 hr of forskolin incubation with no ligand (DMSO control) is set as 100%. n = 6~12 for each dose. Statistical significance is assessed by one-way ANOVA analysis, followed by Dunnett’s multiple comparisons (***p < 0.001, **p < 0.01, *p < 0.05). Dose-response curves are generated using nonlinear regression (curve fit) method in GraphPad.

JH is one of the major insect hormones that regulate vitellogenesis and Vg uptake. It has been speculated that JH acts on a membrane receptor to control the remodeling of the follicular epithelium to allow Vg enter maturing oocytes. Since JH-regulated patency has not yet been investigated in *T. castaneum*, we first tested whether JH can induce patency in isolated ovaries in this insect. Using a scanning electronic microscope (SEM), we observed that the gaps between the follicular epithelial cells (patency) became apparent on day 4 PAE and continued increase with time (Fig. 4A). Interestingly, patency was induced by JH III (but not 20-hydroxy ecdysone, another key insect hormone) in a dose-dependent manner (Fig. 4B,C). These results showed that similar to many other insect species, JH-triggered patency occurs in *T. castaneum*.

To examine the direct interaction between JH and two GPCRs identified in our screens, complete ORFs of TcRh2 and TcD2R were cloned into pCDNA3.1 expression vector and the constructs were transiently transfected into HEK 293 cells together with a 6 × CRE-luciferase reporter construct, which was used to monitor intracellular cAMP levels. Treating these cells with JH III or expressing TcD2R alone showed no effect on cAMP levels. Incubation with 1 μM of forskolin led to ~6-fold increase of cAMP levels, which was partially blocked by adding JH III to TcD2R expressing HEK293 cells (Fig. 4D). In addition, we observed a dose-dependent repression of forskolin-induced cAMP levels by JH III in HEK 293 cells expressing TcD2R but not TcRh2 (Fig. 4E,F). Dopamine also showed similar dose-dependent inhibition of forskolin-induced cAMP in TcD2R expressing HEK293 cells (Fig. 4G). These results suggest that TcD2R is likely a D2 type of dopamine receptor in *T. castaneum*, and it could also mediate the nongenomic action of JH.

## Discussion

The action of JH through a second messenger cascade and membrane receptors (especially during the vitellogenesis and Vg uptake) was proposed a couple of decades ago^1^. However, the proteins that mediate this action remain unknown. The GPCR family, one of the largest membrane receptor protein families, has been suggested as the candidate for mediating the nongenomic action of JH. In the present study, we carried out a series of RNAi screens and functional characterization to identify candidate GPCRs that are involved in female reproduction and Vg uptake in *T. castaneum*. Out of 112 GPCRs examined, we identified two candidates (TcRh2 and TcD2R) that are involved in female fecundity and Vg uptake. Intriguingly, we observed a dose-dependent JH response in TcD2R-expressing HEK293 cells, suggesting that JH could trigger nongenomic actions through a Dopamine D2-like receptor (D2R).

Dopamine D2-like receptor (D2R) belongs to dopamine receptor family, which is prominently expressed in the vertebrate central nervous system. Dopamine receptors are involved in regulation of many biological processes such as motivation, memory, locomotor activity and modulation of neuroendocrine signaling^32^. D2R typically transduces second-messenger signaling via inhibitory G proteins (G_α_i/o), which leads to inhibition of adenylate cyclase and modulation of ion channels^33^. D2R is an alternatively spliced GPCR in mammals^34^. The short splice isoform of D2R receptors is expressed in presynaptic dopaminergic cells and functions as an autoreceptor that controls the release of dopamine. The long splice isoform, however, is mainly expressed in postsynaptic sites and regulates locomotor activities^34^,^35^. In *D. melanogaster*, D2R has been linked to feeding behavior (proboscis extension response)^36^ and D2R RNAi flies show significantly decreased locomotor activity^37^. Interestingly, a recent study suggests that there is a direct link between D2R and JH signaling. The study reported that D2R is expressed in the *corpus allatum* (CA) of female *D. melanogaster* and downregulation of D2R in CA led to decreased JH degradation^38^. Interestingly, the interaction of JH and dopamine was previously reported in the female reproduction of *Drosophila virilis*^39^. Treatment of octopamine, a biogenic amine that is closely related to noradrenaline with dopaminergic effects, inhibits JH III biosynthesis in isolated corpora allata in the cricket, *Gryllus bimaculatus*^40^.

The present study provides further evidence for the cross-talk between JH and Dopamine D2-like receptor. Although we didn’t directly examine this interaction *in vivo*, our RNAi screen and *in vitro* functional assays suggest that D2R may be one of the membrane receptors mediating JH action in ovaries. Several previous studies showed that GPCRs can be activated by steroid hormones *in vitro*. For example, progesterone binds to a novel GPCR cloned from the seatrout ovaries and activated mitogen-activated protein kinase (MAPK) activity and inhibits adenylyl cyclase in a mammalian cell line^41^. An adrenergic-like receptor cloned from *D. melanogaster*, named DmDopEcR, was found to be activated by both the catecholamine DA and ecdysteroids^42^. DmDopEcR is the only membrane receptor to date that is activated by insect ecdysteroids. Our study presents another example that membrane receptor GPCRs can be activated by insect hormone such as JH. However, to prove that JH is the ligand activating D2R, future works including ligand binding assays are required. Furthermore, we need to address whether JH interacts with D2R *in vivo*, especially at ovarian follicle epithelium to regulate Vg uptake. We also notice that in previous studies JH evoked patency mainly by activating G_s_ protein-coupled receptors^11^, instead of G_i_ protein-coupled receptors (inhibitory G protein linked receptor-like D2R). Future works are needed to examine the genetic interaction between JH signaling and D2R in the control of patency and Vg uptake, which will provide us more insights about nongenomic actions of JH in the regulation of insect physiology.

Besides of D2R, we have identified many interesting GPCR candidates that are involved in vitellogenesis and fecundity, for instance, TC011655 (Neuropeptide F receptor), TC001872 (Latrophilin-like receptor), TC000118/Rh2 (Rhodopsin-like receptor), TC012297 (Serotonin receptor 1A-like), and TC007170 (capa receptor-like). Many of these receptors haven not yet been implicated in female reproduction. Taken together, our findings provide new insight into the mechanisms by which GPCRs modulate insect reproductive physiology.

## Additional Information

**How to cite this article**: Bai, H. and Palli, S. R. Identification of G protein-coupled receptors required for vitellogenin uptake into the oocytes of the red flour beetle, *Tribolium castaneum*. *Sci. Rep.* **6**, 27648; doi: 10.1038/srep27648 (2016).

## Supplementary Material

Supplementary Information
