# Supplementary material for: Identification of G protein-coupled receptors required for vitellogenin uptake into the oocytes of the red flour beetle, Tribolium castaneum
Source: Sci Rep. 2016 Jun 9;6:27648. doi: 10.1038/srep27648 (PMC4899757; doi:10.1038/srep27648)
Supplement: Supplementary Information [file srep27648-s1.pdf]

Supplementary Information

Identification of G protein-coupled receptors required for vitellogenin uptake into the oocytes of the red flour beetle, *Tribolium castaneum*

Hua Bai [1,2] and Subba Reddy Palli [2]

[1] Department of Genetics, Development, and Cell Biology, Iowa State University, Ames, IA 50011, USA

[2] Department of Entomology, University of Kentucky, Lexington, KY 40546-0091, USA

Corresponding Authors:

Dr. Hua Bai

Department of Genetics, Development, and Cell Biology, Iowa State University,

Ames, IA 50011, USA, hbai@iastate.edu

Dr. Subba Reddy Palli

Department of Entomology, University of Kentucky S-225 Agricultural

Science Centre North

Lexington, KY 40546-0091

Phone: 859-257-1134, Fax: 859-323-1120, Email: rpalli@uky.edu

Table S1. Effects of GPCR RNAi on female fecundity in *T. castaneum*. The mean number and standard error of eggs laid per female per week are showed (4~10 cohorts, 12~20 pairs of beetles). To compare the mean value of GPCR RNAi versus that of malE control, one-way ANOVA analysis was performed, and followed by Dunnett's multiple comparisons (*** p<0.001, ** p<0.01, * p<0.05, ns-not significant).

Class	Family	Tribolium Official ID	Drosophila homolog	Drosophila symbol	Drosophila full name	Mean	SEM	Significance	Adjusted P Value
Class A: Rhodopsin-like	Biogenic amine	TC007490	CG33517	D2R	Dopamine 2-like receptor	0	0	***	< 0.0001
Class A: Rhodopsin-like	Peptide	TC011655	CG1147	NPFR1	neuropeptide F receptor	0	0	***	< 0.0001
Class B: Secretin receptor-like	Latrophilin-like receptor	TC001872	CG8639	Cirl	-	0	0	***	< 0.0001
Class A: Rhodopsin-like	(Rhod)opsin	TC000118	CG10888	Rh3	Rhodopsin 3	5.99	1.97	***	< 0.0001
Class A: Rhodopsin-like	Biogenic amine	TC012297	CG16720	5-HT1A	Serotonin receptor 1A	7.74	4.51	***	< 0.0001
Class A: Rhodopsin-like	Peptide	TC007170	CG14575	capaR	capa receptor	7.84	2.76	***	< 0.0001
Class D: Atypical	-	TC010568	-	-	-	10.33	2.05	***	< 0.0001
Class A: Rhodopsin-like	Peptide	TC010505	CG8985	DmsR-1	Dromyosuppressin receptor 1	10.41	3.00	***	< 0.0001
Class A: Rhodopsin-like	Biogenic amine	TC011960	CG15113	5-HT1B	Serotonin receptor 1B	10.48	1.83	**	0.0082
Class A: Rhodopsin-like	Biogenic amine	TC011667	CG12073	5-HT7	Serotonin receptor 7	11.00	6.33	***	0.0001
Class A: Rhodopsin-like	Biogenic amine	TC003331	CG12796	CG12796	-	11.06	4.87	*	0.0124
Class A: Rhodopsin-like	Peptide	TC009749	-	-	-	11.16	1.95	***	< 0.0001
Class A: Rhodopsin-like	Peptide	TC001056	CG5811	CG5811	-	11.75	1.82	***	0.0003
Class A: Rhodopsin-like	Protein hormone receptor	TC008163	CG8930	rk	rickets, bursicon receptor	12.35	1.98	*	0.029
Class A: Rhodopsin-like	Peptide	TC015904	CG13229	CG13229	-	12.49	9.21	*	0.0316
Class A: Rhodopsin-like	Peptide	TC011156	CG10823	SIFR	SIFamide receptor	13.07	1.43	**	0.0011
Class C: Metabotropic glutamate receptor-like	GABA-B receptors	TC014995	CG6706	GABA-B-R2	metabotropic GABA-B receptor subtype 2	13.17	1.22	**	0.0013
Class A: Rhodopsin-like	Peptide	TC007986	CG33696	CG33696	-	13.65	4.47	*	0.0104
Class A: Rhodopsin-like	Biogenic amine	TC012597	CG33976	Octbeta2R	Octopamine receptor	13.73	3.95	***	0.0006
Class A: Rhodopsin-like	Peptide	TC011318	CG9918	CG9918	pyrokinin-1 receptor	13.84	4.24	***	0.0002
Class B: Secretin receptor-like	Methuselah-like	TC010567	CG17061	mthl10	methuselah-like 10	13.89	0.84	***	0.0002
Class A: Rhodopsin-like	Peptide	TC014211	-	-	-	14.24	2.13	**	0.0038
Class A: Rhodopsin-like	Peptide	TC004716	CG3171	Tre1	-	14.90	5.05	**	0.0073
Class A: Rhodopsin-like	Protein hormone receptor	TC009127	CG7665	CG7665	GPA2/GPB5 receptor	15.00	2.48	**	0.0081
Class A: Rhodopsin-like	(Rhod)opsin	TC013765	CG4550	Rh1, ninaE	Rhodopsin 1	15.19	1.03	ns	0.1412
Class C: Metabotropic glutamate receptor-like	Glutamate-like receptors	TC013642	CG11144	mGluRA	metabotropic glutamate receptor	15.37	1.37	**	0.0014
Class A: Rhodopsin-like	Peptide	TC005641	-	-	-	15.39	1.21	*	0.0115
Class B: Secretin receptor-like	-	TC012521	CG11895	stan	starry night	15.43	1.55	**	0.004
Class A: Rhodopsin-like	Peptide	TC016363	-	-	-	15.44	1.78	*	0.012
Class A: Rhodopsin-like	Biogenic amine	TC011639	CG3856	Oamb	Octopamine receptor in mushroom bodies	15.48	4.92	*	0.0125
Class A: Rhodopsin-like	Biogenic amine	TC012447	CG9652	DopR	Dopamine receptor	15.56	0.08	ns	0.168
Class A: Rhodopsin-like	Peptide	TC008438	CG32540	CCKLR-17D3	sulfakinin receptor	15.82	1.20	*	0.0169
Class D: Atypical	Frizzled-Smoothened	TC014055	CG17697	fz	frizzled	15.83	2.99	*	0.0171
Class A: Rhodopsin-like	-	TC013650	CG7497	CG7497	-	15.87	2.24	**	0.0064
Class A: Rhodopsin-like	Biogenic amine	TC012598	CG42244	Octbeta3R	Octopamine receptor	15.93	1.40	*	0.0187
Class A: Rhodopsin-like	Peptide	TC003150	CG7395	sNPF-R	short neuropeptide F receptor	15.95	1.84	*	0.0189
Class A: Rhodopsin-like	Biogenic amine	TC011641	CG18208	CG18208	-	15.95	4.20	ns	0.0584
Class B: Secretin receptor-like	Calcitonin receptor-like	TC013321	CG4395	CG4395	-	15.97	2.07	*	0.0192
Class A: Rhodopsin-like	Peptide	TC006805	CG14593	CCHa2r	CCHamide-2 receptor	16.21	1.46	*	0.0237
Class B: Secretin receptor-like	Diuretic hormone receptor	TC012799	CG8422	Dh44-R1	Diuretic hormone 44 receptor 1	16.63	1.91	*	0.0335
Class A: Rhodopsin-like	-	TC016387	-	-	-	16.74	2.11	*	0.0367
Class B: Secretin receptor-like	Calcitonin receptor-like	TC001222	-	-	-	16.74	1.69	*	0.0367
Class A: Rhodopsin-like	-	TC007259	-	-	-	16.87	2.98	*	0.0173
Class A: Rhodopsin-like	-	TC006608	-	-	-	16.89	2.29	ns	0.2994
Class A: Rhodopsin-like	Protein hormone receptor	TC015777	CG34411	CG34411	-	16.96	5.16	ns	0.3084
Class A: Rhodopsin-like	Biogenic amine	TC013982	CG8007	CG42796	-	17.30	4.73	ns	0.1351
Class A: Rhodopsin-like	-	TC015120	-	-	-	17.40	4.42	ns	0.1439
Class A: Rhodopsin-like	Biogenic amine	TC001180	CG13579	CG13579	-	17.41	4.13	ns	0.3655
Class C: Metabotropic glutamate receptor-like	-	TC010857	-	-	-	17.52	1.94	ns	0.0662
Class C: Metabotropic glutamate receptor-like	GABA-B receptors	TC016191	CG15274	GABA-B-R1	metabotropic GABA-B receptor subtype 1	17.67	2.17	ns	0.0743
Class C: Metabotropic glutamate receptor-like	Glutamate-like receptors	TC004727	CG11144	mGluRA	metabotropic glutamate receptor	17.78	3.03	ns	0.08
Class B: Secretin receptor-like	Calcitonin receptor-like	TC008110	-	-	-	17.80	4.83	ns	0.4221
Class A: Rhodopsin-like	Peptide	TC004977	CG6515	Takr86C	Tachykinin-like receptor at 86C	18.04	2.05	ns	0.096
Class A: Rhodopsin-like	Purine	TC012510	CG9753	AdoR	Adenosine receptor	18.16	2.25	*	0.0295
Class A: Rhodopsin-like	Peptide	TC014731	CG34381	CG34381	-	18.50	1.29	ns	0.1302
Class A: Rhodopsin-like	Peptide	TC002068	-	-	-	18.52	2.83	ns	0.1324
Class C: Metabotropic glutamate receptor-like	GABA-B receptors	TC007169	CG3022	GABA-B-R3	metabotropic GABA-B receptor subtype 3	18.69	1.48	ns	0.1478
Class D: Atypical	-	TC012209	-	-	-	18.69	5.36	ns	0.1478
Class A: Rhodopsin-like	Biogenic amine	TC012600	CG6919	oa2	Octopamine receptor 2	18.75	9.00	ns	0.2908
Class A: Rhodopsin-like	Biogenic amine	TC000574	CG18741	DopR2	Dopamine receptor 2	18.80	0.17	ns	0.5827
Class A: Rhodopsin-like	Peptide	TC004565	CG32547	CG32547	-	19.01	3.94	ns	0.18
Class A: Rhodopsin-like	Peptide	TC011171	CG8784	CG8784	pyrokinin-2 receptor	19.09	1.10	ns	0.1878
Class B: Secretin receptor-like	-	TC009527	CG11318	CG11318	-	19.28	3.06	ns	0.2108
Class A: Rhodopsin-like	Biogenic amine	TC013979	CG1056	5-HT2	Serotonin receptor 2	19.38	1.91	ns	0.2237
Class A: Rhodopsin-like	Peptide	TC007687	CG4322	moody	-	19.41	3.93	ns	0.2276
Class A: Rhodopsin-like	Peptide	TC003492	CG6986	Proc-R	Proctolin receptor	19.61	2.31	ns	0.2537
Class A: Rhodopsin-like	Peptide	TC011478	CG13229	CG13229	-	19.62	2.50	ns	0.2552
Class B: Secretin receptor-like	Diuretic hormone receptor	TC002694	CG32843	Dh31-R1	Diuretic hormone 31 receptor 1	19.87	2.39	ns	0.292
Class D: Atypical	Frizzled-Smoothened	TC006527	CG4626	fz4	frizzled 4	19.96	2.49	ns	0.1983
Class A: Rhodopsin-like	Biogenic amine	TC004470	CG4356	mAcR-60C	muscarinic Acetylcholine Receptor 60C	19.97	4.03	ns	0.3077
Class A: Rhodopsin-like	Peptide	TC002497	CG30340	CG30340	-	20.26	1.32	ns	0.3567
Class D: Atypical	Frizzled-Smoothened	TC003407	CG9739	fz2	frizzled 2	20.26	2.95	ns	0.3567
Class A: Rhodopsin-like	Peptide	TC001381	CG2114	FR	FMRFamides receptor	20.65	1.18	ns	0.4297
Class A: Rhodopsin-like	Peptide	TC009772	CG11325	AKHR	Adipokinetic hormone receptor	21.11	2.92	ns	0.385
Class A: Rhodopsin-like	Biogenic amine	TC000298	CG7918	CG7918	-	21.13	2.76	ns	0.7226
Class A: Rhodopsin-like	Peptide	TC002917	CG16752	SPR	Sex peptide receptor,MIP/allatostatin B receptor	21.26	6.65	ns	0.7499
Class A: Rhodopsin-like	Peptide	TC002216	CG33344	CcapR	Crustacean cardioactive peptide receptor	21.57	1.50	ns	0.6257
Class B: Secretin receptor-like	-	TC001376	CG11318	CG11318	-	21.57	3.50	ns	0.6257
Class C: Metabotropic glutamate receptor-like	-	TC013504	CG31660	pog	poor gastrulation	21.57	5.19	ns	0.9638
Class C: Metabotropic glutamate receptor-like	-	TC005523	CG31760	CG31760	-	21.58	1.73	ns	0.3809
Class C: Metabotropic glutamate receptor-like	Glutamate-like receptors	TC004301	CG30361	mtt, DmXR	mangetout, DmX receptor	21.83	2.04	ns	0.6853
Class A: Rhodopsin-like	Biogenic amine	TC004545	CG7485	TyrR,OcR	Octopamine/Tyramine receptor	21.96	4.99	ns	0.9788
Class A: Rhodopsin-like	Peptide	TC009184	-	-	-	22.09	2.18	ns	0.7441
Class A: Rhodopsin-like	Biogenic amine	TC006457	CG18314	DopEcR	Dopamine-Ecdysteroid receptor	22.17	2.25	ns	0.625
Class A: Rhodopsin-like	-	TC003432	-	-	-	22.35	2.25	ns	0.8
Class B: Secretin receptor-like	-	TC013682	-	-	-	22.39	2.73	ns	0.9816
Class B: Secretin receptor-like	Diuretic hormone receptor	TC007104	CG8422	Dh44-R1	Diuretic hormone 44 receptor 1	22.44	3.47	ns	0.5845
Class A: Rhodopsin-like	Biogenic amine	TC012122	CG7431	CG7431	Tyramine receptor	22.57	3.99	ns	0.9503
Class D: Atypical	-	TC002308	CG8285	boss	bride of sevenless	22.62	2.69	ns	0.8515
Class A: Rhodopsin-like	Peptide	TC011320	CG9918	CG9918	pyrokinin-1 receptor	22.75	3.20	ns	0.8747
Class B: Secretin receptor-like	Methuselah-like	TC009370	CG6965	mthl5	methuselah-like 5	23.17	2.66	ns	0.9362
Class A: Rhodopsin-like	Peptide	TC011198	CG7887	Takr99D	Tachykinin-like receptor at 99D	23.27	1.69	ns	0.9474
Class A: Rhodopsin-like	Peptide	TC003151	CG7395	sNPF-R	short neuropeptide F receptor	23.62	3.00	ns	0.8725
Class A: Rhodopsin-like	Peptide	TC012493	CG5911	ETHR	Ecdysis-triggering hormone receptor	23.90	3.56	ns	0.9213
Class A: Rhodopsin-like	Peptide	TC007536	CG42301	CCKLR-17D1	sulfakinin receptor	23.92	3.08	ns	0.98
Class C: Metabotropic glutamate receptor-like	-	TC010500	CG32447	CG32447	-	24.02	3.57	ns	0.9678
Class B: Secretin receptor-like	-	TC016199	-	-	-	24.90	2.38	ns	0.9911
Class A: Rhodopsin-like	Peptide	TC005327	CG30106	CCHa1r	CCHamide-1 receptor	24.97	1.34	ns	0.998

Class D: Atypical	Frizzled-Smoothened	TC005545	CG11561	smo	smoothened	25.54	2.95	ns	0.9983
Class A: Rhodopsin-like	Peptide	TC013945	CG33344	CcapR	Crustacean cardioactive peptide receptor	25.56	5.93	ns	0.9983
Class A: Rhodopsin-like	Peptide	TC010818	CG13995	CG13995	-	25.60	3.89	ns	0.9989
Class A: Rhodopsin-like	-	TC003453	-	-	-	26.67	3.09	ns	0.9989
Class A: Rhodopsin-like	Protein hormone receptor	TC009575	CG7665	CG7665	GPA2/GPB5 receptor	26.83	4.30	ns	0.9993
Class B: Secretin receptor-like	-	TC010267	CG12370	CG12370	-	28.12	2.46	ns	0.9996
Class A: Rhodopsin-like	Peptide	TC012842	CG13702	AlCR2	allatostatin C receptor 2	28.12	0.82	ns	0.9996
Class B: Secretin receptor-like	-	TC013091	-	-	-	28.23	4.62	ns	0.9996
Class B: Secretin receptor-like	Methuselah-like	TC010656	CG4521	mthl1	methuselah-like 1	28.37	4.29	ns	0.9996
Class A: Rhodopsin-like	Peptide	TC002524	CG33639	CG33639	-	28.63	2.38	ns	0.9997
Class A: Rhodopsin-like	Peptide	TC006482	-	-	-	29.28	2.45	ns	0.9999
Class B: Secretin receptor-like	-	TC000764	-	-	-	29.28	2.05	ns	0.9999
Class C: Metabotropic glutamate receptor-like	Glutamate-like receptors	TC001106	CG11144	mGluRA	metabotropic glutamate receptor	30.85	2.93	ns	0.9996
Class A: Rhodopsin-like	Peptide	TC001245	CG11325	AKHR	Adipokinetic hormone receptor	33.60	3.75	ns	0.9985
malE (control)	/	/	/	/	/	29.71	3.58	/	/

Table S2. Effects of GPCR RNAi on larva hatch rate in *T. castaneum*. The mean percentage of hatch rate is showed (4~10 cohorts, 12~20 pairs of beetles). To compare the mean value of GPCR RNAi versus that of malE control, one-way ANOVA analysis was performed, and followed by Dunnett's multiple comparisons (*** p<0.001, ** p<0.01, * p<0.05, ns-not significant).

Class	Family	Tribolium Official ID	Drosophila homolog	Drosophila symbol	Drosophila full name	Mean	SEM	Significance	Adjusted P Value
Class A: Rhodopsin-like	Peptide	TC004716	CG3171	Tre1	-	0	0	***	< 0.0001
Class C: Metabotropic glutamate receptor-like	GABA-B receptors	TC007169	CG3022	GABA-B-R3	metabotropic GABA-B receptor subtype 3	0	0	***	< 0.0001
Class C: Metabotropic glutamate receptor-like	-	TC010857	-	-	-	0	0	***	< 0.0001
Class A: Rhodopsin-like	Peptide	TC013945	CG33344	CcapR	Crustacean cardioactive peptide receptor	0.36	0.36	***	< 0.0001
Class A: Rhodopsin-like	Peptide	TC014211	-	-	-	0.81	0.81	***	< 0.0001
Class D: Atypical	Frizzled-Smoothened	TC005545	CG11561	smo	smoothened	0.91	0.58	***	< 0.0001
Class A: Rhodopsin-like	Biogenic amine	TC012297	CG16720	5-HT1A	Serotonin receptor 1A	1.40	0.92	***	< 0.0001
Class A: Rhodopsin-like	Peptide	TC003150	CG7395	sNPF-R	short neuropeptide F receptor	1.49	1.49	***	< 0.0001
Class D: Atypical	Frizzled-Smoothened	TC003407	CG9739	fz2	frizzled 2	2.12	0.82	***	< 0.0001
Class A: Rhodopsin-like	Peptide	TC009184	-	-	-	3.04	1.14	***	< 0.0001
Class A: Rhodopsin-like	Biogenic amine	TC012600	CG6919	oa2	Octopamine receptor 2	3.59	2.12	***	< 0.0001
Class A: Rhodopsin-like	Biogenic amine	TC011639	CG3856	Oamb	Octopamine receptor in mushroom bodies	3.62	2.21	***	< 0.0001
Class A: Rhodopsin-like	-	TC013650	CG7497	CG7497	-	3.68	1.97	***	< 0.0001
Class D: Atypical	Frizzled-Smoothened	TC014055	CG17697	fz	frizzled	3.83	2.21	***	< 0.0001
Class A: Rhodopsin-like	Purine	TC012510	CG9753	AdoR	Adenosine receptor	3.39	1.62	***	< 0.0001
Class C: Metabotropic glutamate receptor-like	-	TC005523	CG31760	CG31760	-	4.43	1.40	***	< 0.0001
Class A: Rhodopsin-like	Peptide	TC004565	CG32547	CG32547	-	4.39	3.39	***	< 0.0001
Class D: Atypical	-	TC010568	-	-	-	5.18	2.20	***	< 0.0001
Class A: Rhodopsin-like	Protein hormone receptor	TC009127	CG7665	CG7665	GPA2/GPB5 receptor	6.05	3.66	***	< 0.0001
Class A: Rhodopsin-like	Peptide	TC011318	CG9918	CG9918	pyrokinin-1 receptor	6.08	2.57	***	< 0.0001
Class A: Rhodopsin-like	Peptide	TC007536	CG42301	CCKLR-17D1	sulfakinin receptor	8.40	5.04	***	< 0.0001
Class B: Secretin receptor-like	Diuretic hormone receptor	TC007104	CG8422	Dh44-R1	Diuretic hormone 44 receptor 1	10.05	4.88	***	< 0.0001
Class B: Secretin receptor-like	Methuselah-like	TC010656	CG4521	mthl1	methuselah-like 1	10.23	4.73	***	< 0.0001
Class B: Secretin receptor-like	Methuselah-like	TC009370	CG6965	mthl5	methuselah-like 5	10.67	6.77	***	< 0.0001
Class A: Rhodopsin-like	Peptide	TC007687	CG4322	moody	-	13.72	6.83	***	< 0.0001
Class A: Rhodopsin-like	Peptide	TC003151	CG7395	sNPF-R	short neuropeptide F receptor	12.13	4.58	***	< 0.0001
Class A: Rhodopsin-like	Peptide	TC012493	CG5911	ETHR	Ecdysis-triggering hormone receptor	12.45	8.83	***	< 0.0001
Class A: Rhodopsin-like	Peptide	TC001245	CG11325	AKHR	Adipokinetic hormone receptor	15.52	7.67	***	0.0001
Class A: Rhodopsin-like	(Rhod)opsin	TC000118	CG10888	Rh3	Rhodopsin 3	17.58	9.41	***	< 0.0001
Class A: Rhodopsin-like	Peptide	TC011320	CG9918	CG9918	pyrokinin-1 receptor	23.91	8.56	***	0.0003
Class A: Rhodopsin-like	Peptide	TC007986	CG33696	CG33696	-	25.78	15.55	**	0.0042
Class A: Rhodopsin-like	Peptide	TC009749	-	-	-	37.14	12.57	**	0.0057
Class A: Rhodopsin-like	Biogenic amine	TC011960	CG15113	5-HT1B	Serotonin receptor 1B	36.21	7.98	ns	0.2261
Class A: Rhodopsin-like	-	TC006608	-	-	-	39.34	10.48	ns	0.3516
Class A: Rhodopsin-like	Peptide	TC001056	CG5811	CG5811	-	40.45	15.83	ns	0.0755
Class A: Rhodopsin-like	-	TC016387	-	-	-	41.88	9.05	ns	0.1069
Class B: Secretin receptor-like	-	TC001376	CG11318	CG11318	-	42.63	14.48	ns	0.127
Class A: Rhodopsin-like	(Rhod)opsin	TC013765	CG4550	Rh1, ninaE	Rhodopsin 1	43.47	0.18	ns	0.5718
Class A: Rhodopsin-like	Biogenic amine	TC001180	CG13579	CG13579	-	43.99	1.67	ns	0.603
Class B: Secretin receptor-like	Methuselah-like	TC010567	CG17061	mthl10	methuselah-like 10	51.12	14.26	ns	0.3465
Class A: Rhodopsin-like	Peptide	TC010505	CG8985	DmsR-1	Dromyosuppressin receptor 1	49.18	14.17	ns	0.2293
Class A: Rhodopsin-like	-	TC003453	-	-	-	47.21	2.84	ns	0.3203
Class A: Rhodopsin-like	Peptide	TC005327	CG30106	CCHa1r	CCHamide-1 receptor	48.76	2.62	ns	0.4165
Class A: Rhodopsin-like	Peptide	TC011156	CG10823	SIFR	SIFamide receptor	49.71	16.55	ns	0.4827
Class A: Rhodopsin-like	Biogenic amine	TC004545	CG7485	TyrR,OcR	Octopamine/Tyramine receptor	50.18	50.18	ns	0.9285
Class A: Rhodopsin-like	Biogenic amine	TC000574	CG18741	DopR2	Dopamine receptor 2	50.46	43.00	ns	0.9374
Class A: Rhodopsin-like	Biogenic amine	TC012447	CG9652	DopR	Dopamine receptor	51.80	28.21	ns	0.9662
Class B: Secretin receptor-like	-	TC000764	-	-	-	53.11	1.87	ns	0.7453
Class B: Secretin receptor-like	Diuretic hormone receptor	TC012799	CG8422	Dh44-R1	Diuretic hormone 44 receptor 1	53.48	16.34	ns	0.7727
Class B: Secretin receptor-like	-	TC012521	CG11895	stan	starry night	53.57	9.27	ns	0.6472
Class B: Secretin receptor-like	-	TC013091	-	-	-	53.80	20.75	ns	0.7966
Class A: Rhodopsin-like	Peptide	TC015904	CG13229	CG13229	-	54.29	26.76	ns	0.9824
Class C: Metabotropic glutamate receptor-like	Glutamate-like receptors	TC013642	CG11144	mGluRA	metabotropic glutamate receptor	59.23	15.21	ns	0.9608
Class D: Atypical	Frizzled-Smoothened	TC006527	CG4626	fz4	frizzled 4	55.18	13.80	ns	0.7819
Class A: Rhodopsin-like	Biogenic amine	TC011667	CG12073	5-HT7	Serotonin receptor 7	56.07	19.18	ns	0.9301
Class A: Rhodopsin-like	Peptide	TC002068	-	-	-	57.12	7.43	ns	0.9638
Class A: Rhodopsin-like	Peptide	TC006482	-	-	-	58.46	7.60	ns	0.9803
Class A: Rhodopsin-like	-	TC003432	-	-	-	58.95	5.67	ns	0.9818
Class C: Metabotropic glutamate receptor-like	Glutamate-like receptors	TC004301	CG30361	mtt, DmXR	mangetout, DmX receptor	60.77	7.48	ns	0.9919
Class A: Rhodopsin-like	Peptide	TC011478	CG13229	CG13229	-	64.19	9.28	ns	0.9986
Class B: Secretin receptor-like	Calcitonin receptor-like	TC001222	-	-	-	64.22	19.78	ns	0.9986
Class C: Metabotropic glutamate receptor-like	Glutamate-like receptors	TC001106	CG11144	mGluRA	metabotropic glutamate receptor	64.40	6.39	ns	0.9986
Class A: Rhodopsin-like	Biogenic amine	TC013979	CG1056	5-HT2	Serotonin receptor 2	64.50	7.16	ns	0.9986
Class A: Rhodopsin-like	Biogenic amine	TC012122	CG7431	CG7431	Tyramine receptor	65.52	5.31	ns	0.999
Class A: Rhodopsin-like	Peptide	TC008438	CG32540	CCKLR-17D3	sulfakinin receptor	65.62	7.53	ns	0.9988
Class A: Rhodopsin-like	Biogenic amine	TC003331	CG12796	CG12796	-	67.02	3.53	ns	0.9993
Class D: Atypical	-	TC012209	-	-	-	67.04	5.21	ns	0.999
Class C: Metabotropic glutamate receptor-like	Glutamate-like receptors	TC004727	CG11144	mGluRA	metabotropic glutamate receptor	67.48	8.99	ns	0.9991
Class A: Rhodopsin-like	Peptide	TC006805	CG14593	CCHa2r	CCHamide-2 receptor	67.98	8.97	ns	0.9992
Class A: Rhodopsin-like	Peptide	TC003492	CG6986	Proc-R	Proctolin receptor	68.95	8.12	ns	0.9993
Class A: Rhodopsin-like	Biogenic amine	TC011641	CG18208	CG18208	-	70.21	8.15	ns	0.9996
Class B: Secretin receptor-like	Diuretic hormone receptor	TC002694	CG32843	Dh31-R1	Diuretic hormone 31 receptor 1	70.91	10.65	ns	0.9996
Class C: Metabotropic glutamate receptor-like	-	TC010500	CG32447	CG32447	-	71.75	7.24	ns	0.9996
Class A: Rhodopsin-like	Peptide	TC002524	CG33639	CG33639	-	72.03	7.98	ns	0.9997
Class B: Secretin receptor-like	-	TC016199	-	-	-	72.51	6.22	ns	0.9997
Class A: Rhodopsin-like	Peptide	TC014731	CG34381	CG34381	-	72.70	1.91	ns	0.9997
Class A: Rhodopsin-like	Peptide	TC004977	CG6515	Takr86C	Tachykinin-like receptor at 86C	73.32	7.81	ns	0.9998
Class D: Atypical	-	TC002308	CG8285	boss	bride of sevenless	73.45	7.65	ns	0.9999
Class A: Rhodopsin-like	Biogenic amine	TC006457	CG18314	DopEcR	Dopamine-Ecdysteroid receptor	73.62	5.70	ns	0.9999
Class A: Rhodopsin-like	Biogenic amine	TC004470	CG4356	mAcR-60C	muscarinic Acetylcholine Receptor 60C	74.28	13.09	ns	> 0.9999
Class A: Rhodopsin-like	Biogenic amine	TC013982	CG8007	CG42796	-	74.43	12.42	ns	> 0.9999
Class A: Rhodopsin-like	Peptide	TC011198	CG7887	Takr99D	Tachykinin-like receptor at 99D	75.17	7.27	ns	> 0.9999
Class A: Rhodopsin-like	Peptide	TC001381	CG2114	FR	FMRFamides receptor	76.03	6.81	ns	0.9999
Class A: Rhodopsin-like	Peptide	TC007170	CG14575	capaR	capa receptor	76.19	4.53	ns	0.9999
Class B: Secretin receptor-like	Calcitonin receptor-like	TC008110	-	-	-	76.30	6.66	ns	0.9999
Class B: Secretin receptor-like	Calcitonin receptor-like	TC013321	CG4395	CG4395	-	77.00	6.75	ns	0.9998
Class B: Secretin receptor-like	-	TC009527	CG11318	CG11318	-	77.49	5.70	ns	0.9997
Class A: Rhodopsin-like	Biogenic amine	TC012597	CG33976	Octbeta2R	Octopamine receptor	77.99	6.77	ns	0.9996
Class A: Rhodopsin-like	Peptide	TC002917	CG16752	SPR	Sex peptide receptor,MIP/allatostatin B receptor	80.06	5.98	ns	0.9995
Class A: Rhodopsin-like	Peptide	TC002497	CG30340	CG30340	-	80.26	3.91	ns	0.9994
Class A: Rhodopsin-like	Peptide	TC011171	CG8784	CG8784	pyrokinin-2 receptor	80.64	3.59	ns	0.9994
Class C: Metabotropic glutamate receptor-like	GABA-B receptors	TC016191	CG15274	GABA-B-R1	metabotropic GABA-B receptor subtype 1	80.82	9.38	ns	0.9993
Class A: Rhodopsin-like	Biogenic amine	TC000298	CG7918	CG7918	-	81.37	12.24	ns	0.9993
Class A: Rhodopsin-like	Peptide	TC016363	-	-	-	81.95	3.88	ns	0.9992
Class A: Rhodopsin-like	Biogenic amine	TC012598	CG42244	Octbeta3R	Octopamine receptor	82.21	3.84	ns	0.9991
Class A: Rhodopsin-like	-	TC015120	-	-	-	82.38	5.81	ns	0.9992
Class A: Rhodopsin-like	Peptide	TC005641	-	-	-	85.16	6.45	ns	0.9986
Class A: Rhodopsin-like	Peptide	TC009772	CG11325	AKHR	Adipokinetic hormone receptor	85.92	3.54	ns	0.9983
Class A: Rhodopsin-like	Protein hormone receptor	TC015777	CG34411	CG34411	-	85.95	2.14	ns	0.999
Class C: Metabotropic glutamate receptor-like	GABA-B receptors	TC014995	CG6706	GABA-B-R2	metabotropic GABA-B receptor subtype 2	86.85	5.18	ns	0.9983
Class A: Rhodopsin-like	-	TC007259	-	-	-	87.39	1.89	ns	0.998
Class A: Rhodopsin-like	Peptide	TC002216	CG33344	CcapR	Crustacean cardioactive peptide receptor	88.19	3.12	ns	0.998
Class C: Metabotropic glutamate receptor-like	-	TC013504	CG31660	pog	poor gastrulation	90.64	2.98	ns	0.9983
Class B: Secretin receptor-like	-	TC013682	-	-	-	95.03	5.54	ns	0.9836

Class B: Secretin receptor-like	-	TC010267	CG12370	CG12370	-	96.04	2.30	ns	0.9813
Class A: Rhodopsin-like	Peptide	TC012842	CG13702	AlCR2	allatostatin C receptor 2	94.06	5.94	ns	0.9914
Class A: Rhodopsin-like	Peptide	TC010818	CG13995	CG13995	-	96.76	3.24	ns	0.9797
Class A: Rhodopsin-like	Protein hormone receptor	TC009575	CG7665	CG7665	GPA2/GPB5 receptor	100.00	0.00	ns	0.9174
Class A: Rhodopsin-like	Protein hormone receptor	TC008163	CG8930	rk	rickets, bursicon receptor	100.00	0.00	ns	0.9174
malE (control)	/	/	/	/	/	74.93	4.03	/	/
